# Molecular mechanism of cardol, isolated from *Trigona incisa* stingless bee propolis, induced apoptosis in the SW620 human colorectal cancer cell line

**DOI:** 10.1186/s40360-017-0139-4

**Published:** 2017-05-04

**Authors:** Paula Mariana Kustiawan, Kriengsak Lirdprapamongkol, Tanapat Palaga, Songchan Puthong, Preecha Phuwapraisirisan, Jisnuson Svasti, Chanpen Chanchao

**Affiliations:** 1 Program in Biotechnology, Faculty of Science, Chulalongkorn University, 254 Phayathai Road, Bangkok, 10330 Thailand; 2 Laboratory of Biochemistry, Chulabhorn Research Institute, 54 Kamphaeng Phet 6 Road, Bangkok, 10210 Thailand; 3 Department of Microbiology, Faculty of Science, Chulalongkorn University, 254 Phayathai Road, Bangkok, 10330 Thailand; 4 Institute Biotechnology and Genetic Engineering, Chulalongkorn University, 254, Phayathai Road, Bangkok, 10330 Thailand; 5 Department of Chemistry, Faculty of Science, Chulalongkorn University, 254 Phayathai Road, Bangkok, 10330 Thailand; 6 Department of Biology, Faculty of Science, Chulalongkorn University, 254 Phayathai Road, Bangkok, 10330 Thailand

**Keywords:** Apoptosis, Cardol, Mitochondrial apoptotic pathway, Propolis, SW620 cancer cells

## Abstract

**Background:**

Cardol is a major bioactive constituent in the *Trigona incisa* propolis from Indonesia, with a strong in vitro antiproliferative activity against the SW620 colorectal adenocarcinoma cell line (IC_50_ of 4.51 ± 0.76 μg/mL). Cardol induced G_0_/G_1_ cell cycle arrest and apoptotic cell death. The present study was designed to reveal the mechanism of cardol’s antiproliferative effect and induction of apoptosis.

**Methods:**

Changes in cell morphology were observed by light microscopy. To determine whether the mitochondrial apoptotic pathway was involved in cell death, caspase-3 and caspase-9 activities, western blot analysis, mitochondrial membrane potential, and intracellular reactive oxygen species (ROS) levels were assayed.

**Results:**

Changes in the cell morphology and the significantly increased caspase-3 and caspase-9 activities, plus the cleavage of pro-caspase-3, pro-caspase-9 and PARP, supported that cardol caused apoptosis in SW620 cells within 2 h after treatment by cardol. In addition, cardol decreased the mitochondrial membrane potential while increasing the intracellular ROS levels in a time- and dose-dependent manner. Antioxidant treatment supported that the cardol-induced cell death was dependent on ROS production.

**Conclusion:**

Cardol induced cell death in SW620 cells was mediated by oxidative stress elevation and the mitochondrial apoptotic pathway, and these could be the potential molecular mechanism for the antiproliferative effect of cardol.

## Background

Several species of stingless bees (*Trigona* spp.) are native to Kebun Raya Unmul Samarinda (University of Mulawarman Samarinda botanical garden), East Kalimantan province, Indonesia, and play an important role in pollination. However, it is becoming increasingly hard to find them due to the effect of deforestation and forest fragmentation [1].

Bee products from some of the stingless bee species in that area have been screened for their in vitro antiproliferative activity against different human cancer-derived cell lines, and the propolis from *Trigona incisa* was found to be the most promising of them as a source of antiproliferative agents [2]. Subsequently, cardol or 5-pentadecyl resorcinol (C_21_H_36_O_2_), was reported to be the major antiproliferative compound isolated from *T. incisa* [3], although high amounts of essential oils, diterpenes, triterpenes and some prenylated derivatives of p-coumaric acid were also present [4]. Overall, the major bioactive compounds in the different propolis types were phenolic acids and flavonols [5].

Cardol is widely found in members of the *Anarcadiaceae* plant family, and so the resin (etc) from these plants is the likely source of it in propolis. Interestingly, cardol has also been reported in *Apis mellifera* propolis in Thailand [6]. However, it is common to find the same active compounds in propolis from different bee species or in neighboring countries in the same tropical region due to the shared species of *Anarcadiaceae* plants from which the bees harvest the resin (amongst other sources) to make the propolis.

Cardol is a related group of compounds based upon alkyresorcinol with a variable long chain side. Alkylresorcinols have been reported to have many bioactivities, such as antiparasitic, anticancer, antifungal, antimicrobial and antioxidant activities. The long 5-alkyl side chain containing cardol (C15:3) is a unique xanthine oxidase inhibitor without any pro-oxidant effects [7], and has an inhibition concentration at 50% (IC_50_) value for superoxide anion generation of 115 ± 10 μM. The cardol with a medium length chain (C10:0) also inhibited superoxide anion generation, but the small length chain cardol (C5:0) and resorcinol (no 5-alkyl side chain) did not. Thus, the 5-alkyl side chain seems to play an important role in eliciting the xanthine oxidase inhibitor activity that then inhibits superoxide anion generation by binding cooperatively to the enzyme [8]. Cardol has been reported to exhibit antiprotozoal activity against *Leishmania donovani* (IC_50_ = 22 mM) and *Trypanosoma brucei* (IC_50_ = 13 mM), but not against *Plasmodium falciparum*. Cardol showed a very low antioxidant activity with no in vitro antiproliferative activity to all tested cell lines up to the highest tested concentration of 10 mg/mL (27–31 mM) [9]. The C15:3 cardol, (2),5-[80(Z),110(Z),140-penta-decatrienyl]resorcinol, isolated from cashew nuts was a specific inhibitor for the superoxide anion generation catalysed by xanthine oxidase [10].

The potential composition of propolis, including its bioactivity, depends mainly on the biogeography since it is affected by the species and distribution of the plant sources as well as the bee species [11]. For example, the crude ethanol extract of propolis from the stingless bee *Tetragonisca fiebrigi* in Brazil had a good radical scavenging activity, as determined by the 2,2’-azino-bis(3-ethylbenzothiazoline-6-sulphonic acid) assay, and an antiproliferative (necrosis-inducing) effect on the K562 erythroleukemia cell line, which might be of benefit in the control of cancers that are resistant to conventional chemotherapy or apoptosis [12]. Furthermore, doxorubicin, an effective anticancer drug, can impair testicular function leading to infertility [13]. With the increase in cancer resistance to standard chemotherapeutic drugs as well as their undesired side-effects, finding an alternative agent has always been necessary for cancer treatment. Cardol has become of interest since it can induce early apoptosis in human cancer cell lines [3], but the mechanism of its antiproliferative effect and induction of apoptosis, including in the sensitive SW620 colorectal cancer cells, has not been evaluated.

In this research, the cell morphology of cardol-treated SW620 cells was observed. In order to ascertain any involvement of the mitochondrial apoptotic pathway, the activity of caspase-3 and -9 were assayed. In addition, mitochondrial membrane polarization and the level of intracellular reactive oxygen species (ROS) were also measured.

## Methods

### Cardol

Cardol (5-pentadecyl resorcinol) was purified from *T. incisa* propolis collected from Mulawarman University Botanical Garden, Samarinda, East Kalimantan, Indonesia in 2013 as previously reported [3]. Briefly, crude methanol extract of *T. incisa* propolis was partitioned with n-hexane, ethyl acetate and methanol. By MTT assay, an active sample was further purified by silica gel quick column, absorption and size exclusion chromatography, respectively. By thin layer chromatography, the pure and most active sample was selected. Next, the ^1^H-NMR and ^13^C-NMR analysis was used for chemical structure. Later, the chemical structure of the most active compound was found to be orange solid; DT46-3; ^1^H NMR (CDCl_3_, 400 MHz) δ_H_ 6.17 (2H, d, *J* = 2.0 Hz, H-4, and H-6), 6.10 (1H, s, H-2), 5.28 (2H, m, olefinic proton), 2.39 (2H, t, *J* = 7.6 Hz, H-1'), 1.95 (4H, br s), 1.48 (2H, br s), 1.18–1.25 (38H, br s), 0.82 (3H, t, *J* = 6.8 Hz); ^13^C NMR (CDCl_3_, 100 MHz) δ_C_ 156.5, 146.2, 129.9, 129.8, 108.0, 100.1, 35.8, 31.9, 31.1, 29.8, 29.7, 29.7, 29.6, 29.5, 29.5, 29.3, 27.2, 26.9, 22.3, 14.0; ESIMS *m/z* [M + H]^+^ 459. This NMR spectrum analysis could reveal a structure to be C_31_H_54_O_2_, an isomer of cardol.

### Cell culture

SW620 (ATCC no. CCL 227, Rockville, MD), a human colorectal adenocarcinoma derived cell line, was used in this study at passage 50–200. The cells were seeded at 10^5^ cells per 25-cm^2^ flask in 5 mL of complete medium (CM; RPMI 1640 medium containing 5% (v/v) fetal calf serum). In all cases cells were cultured at 37 °C in a humidified atmosphere of 5% CO_2_. For routine cultures, cells were passaged when at 80–90% confluency and the medium was changed every 3–4 d.

### Cell proliferation/viability determination using the 3-(4,5-dimethyl-thiazol-2-yl)2,5-diphenyl-tetrazolium bromide (MTT) assay

When the SW620 cells reached 80–90% confluency, they were harvested by removing the CM, washing with 1 mL of cold phosphate buffered saline (PBS; 137 mM NaCl, 2.7 mM KCl, 10.6 mM Na_2_HPO_4_, and 0.7 mM KH_2_PO_4_, pH 7.4), and then incubating with 1 mL of 0.05% (w/v) trypsin for 2 min at 37 °C. Cells were dissociated by mixing gently prior to harvesting by centrifugation at 5,000x g for 5 min at room temperature (RT). The cell pellet was resuspended in 1 mL of new CM and a 10 μL aliquot was mixed with an equal volume of tryptophan blue and the number and viability of the cells were counted using a hematocytometer. The remaining cell suspension was adjusted with CM to 2.5 x 10^4^ cells/mL and 198 μL was added per well of a 96-well plate and incubated at 37 °C for 24 h. The cultured cells were then treated with cardol at a final concentration of 0 (control), 1, 0.1, 0.01 or 0.001 μg/mL, all in dimethylsulfoxide (DMSO) at a final concentration of 0.1% (v/v) in triplicate wells per condition. Doxorubicin (Sigma-Aldrich, St. Louis, MO) at a final concentration of 0.5 μg/mL was used as the positive control. The treated cells were cultured for 48 h prior to the addition of 10 μL of 5 mg/mL MTT (Sigma-Aldrich, St. Louis, MO) solution into each well and incubated for 4 h. The media was then removed and a mixture of DMSO (150 μL) and glycine (25 μL) was added and aspirated to dissolve the formed formazan crystals prior to reading the absorbance at 540 nm using a microplate reader (Multiskan FC, Thermo Scientific).

The relative cell viability (%) was calculated from % relative cell viability = [(Abs of sample – Abs of blank)/(Abs of control – Abs of blank)] x 100, where Abs stands for the absorbance at 540 nm. The IC_50_ value was estimated from the plot of cardol concentration against the relative cell viability.

### Cell morphology

SW620 cells were seeded at 8 x 10^5^ cells/mL CM/well in 12-well plates and treated with 0 (control), 8 and 14 μg/mL cardol and incubated for 72 h. The cell morphology was observed after 0, 2, 4, 6, 24, 48 and 72 h incubation using an inverted light microscope (Olympus) connected to a digital camera (Canon).

### Caspase activity assay

SW620 cells were assayed for caspase-3 and -9 activation using the Caspase-Glo assay kits (Catalog # G8091 and G8211, Promega, Madison, WI). SW620 cells were seeded at 8 x 10^3^ cells/100 μL CM in each well of a 96-well plate for 24 h. The medium was then changed to fresh CM supplemented with cardol at 0 (control), 8 and 14 μg/mL and further incubated for the indicated time (0, 15, 30, 45 and 60 min). Each respective caspase acitivity was then assayed according to the manufacturer’s instructions. The luminescence of each sample was measured in a plate-reading luminometer (Thermo Labsystems).

### Western blot analysis

SW620 cells were cultured in 6-well plates at an initial concentration of 8 x 10^3^ cells/well for 24 h. The cells, split into four groups were then treated with (i) DMSO at 0.1% (v/v) (control), (ii) cardol at 8 μg/mL, (iii) cardol at 14 μg/mL and (iv) doxorubicin at 1.01 μg/mL (positive control) and incubated for 2, 4, 6 and 24 h. After the indicated time, the media was removed, cells were rinsed with cold PBS, scraped and then lysed in radio-immunoprecipitation assay buffer (Sigma-Aldrich, St. Louis, MO) containing protease and phosphatase inhibitor cocktail (catalog # 78440, Thermo Scientific, Rockford, IL). The cell lysate was centrifuged at 10,000 x g, 4 °C for 5 min and the supernatant was harvested. The protein concentration was measured by the Bradford assay. Each 10 μg protein portion was fractionated by sodium dodecyl sulfate polyacrylamide gel electrophoresis (SDS-PAGE) with a 10% (w/v) separating gel and 7.5% (w/v) stacking gel at 25 mA for approximately 1 h. The resolved proteins were then transferred to a polyvinylidenediflouride membrane (7 x 9 cm) by electroblotting at 100 V for 1.5 h. The membrane was retrived and soaked/washed with Tris-buffered saline (TBS) containing 0.1% (v/v) Tween 20 (TBST), blocked with TBST containing 3% (w/v) bovine serum albumin (TBSTB) for 1 h at RT and then probed with the indicated primary antibody against PARP (1: 1,000), caspase-3 (1: 1,000), caspase-9 (1: 1,000) or β-actin (1: 10,000), in TBSTB at 4 °C. The membrane was then washed three times in TBST for 10 min and probed with the relevant horseradish peroxidase-conjugated anti-mouse (1: 10,000) or anti-rabbit (1: 5,000) secondary antibody (Glostrup, Denmark) in TBSTB for 1 h. The membrane was then washed in TBST before visualization using the Western Bright ECL reagent (Catalog # RPN2232, GE Healthcare, England) and Image Quant LAS 4,000 mini (GE Healthcare, Life Sciences).

### Mitochondrial membrane potential assay

The mitochondrial membrane polarization was measured using a JC-10 mitochondrial membrane potential assay kit (catalog # ab112134, Abcam, Cambridge, MA) according to the manufacturer’s instructions. SW620 cells were seeded (8 x 10^4^ cells) in 100 μL of CM per well of a 96-well black microplate (tissue culture microplate with black wall and clear bottom). After overnight incubation, the cells were treated with (final concentrations): (i) DMSO at 0.1% (v/v), (ii) cardol at 8 μg/mL and (iii) cardol at 14 μg/mL. The fluorescence intensity was evaluated after 0, 0.5, 1, 1.5 and 2 h using excitation/emission wavelengths at 490/520 nm for the monomeric form found in the cytoplasm, and excitation/emission wavelengths at 540/590 nm for the aggregated form found in the mitochondrial matrix. The mitochondrial membrane potential was indicated by the ratio of aggregated to monomeric forms.

### Measurement of intracellular ROS

The average ROS level inside the SW620 cells was measured using a fluorescent probe. SW620 cells were seeded in each well of 96-well plates at an initial concentration of 8 x 10^3^ cells/well in CM and cultured for 24 h. The cells were then washed twice with 100 μL of 1 x Hank’s buffer salt solution (HBSS, catalog # 14180046, Gibco, Carlsbad, CA) before dihydroethidium (DHE, catalog # D7008, Sigma-Aldrich, St. Louis, MO), dissolved in HBSS to 20 mM, was then added to the incubated cells to a final concentration of 10 mM each and incubated for 30 min. The medium was removed, the cells washed twice with 100 μL HBSS and then subjected to cardol treatment at 0 (control), 8 and 14 μg/mL in HBSS at 37 °C for 2 h. The fluorescence was measured at 0, 0.5, 1, 1.5 and 2 h after initiation of the treatment with a microplate reader, at an excitation/emission wavelength of 495/595 nm for DHE.

### N-acetyl cysteine (NAC) treatment

To ascertain whether the ROS elevation induced by cardol is a molecular mechanism that underlies the antiproliferative effect of the compound, N-acetyl cysteine (NAC, Sigma-Aldrich, St. Louis, MO) was used to reduce the oxidative stress in the treated cells. SW620 cancer cells (5 x 10^3^ cells) were seeded in 100 μL of CM in each well of 96-well plates and incubated for 24 h prior to replacing the medium with 100 μL of fresh RPMI without FCS and incubating for 2 h. The cells, split into six groups, were then treated with (final concentration in 200 μL) (i) DMSO at 0.1% (v/v) as a control, (ii) NAC at 10 mM, (iii) cardol at 8 μg/mL, (iv) cardol at 14 μg/mL, (v) NAC at 10 mM plus cardol at 8 μg/mL and (vi) NAC at 10 mM plus cardol at 14 μg/mL, and then incubated for 24 or 72 h. At the designated time point the relative number of viable cells was evaluated by the MTT assay as above, measuring the final absorbance at 540 nm on a microplate reader and standardized to that of the control (set as 100%).

### Data analysis

Data are presented as the mean ± one standard error (SE), derived from the indicated number of independent repeats. The significance of any difference between means was tested using one-way analysis of variance (ANOVA) followed by Tukey’s test of multiple comparisons, accepting significance at the *p* < 0.05 level. All analyses were performed using the SPSS program version 19.0.

## Results

### Effect of cardol on the SW620 cell morphology

**Fig. 1:**
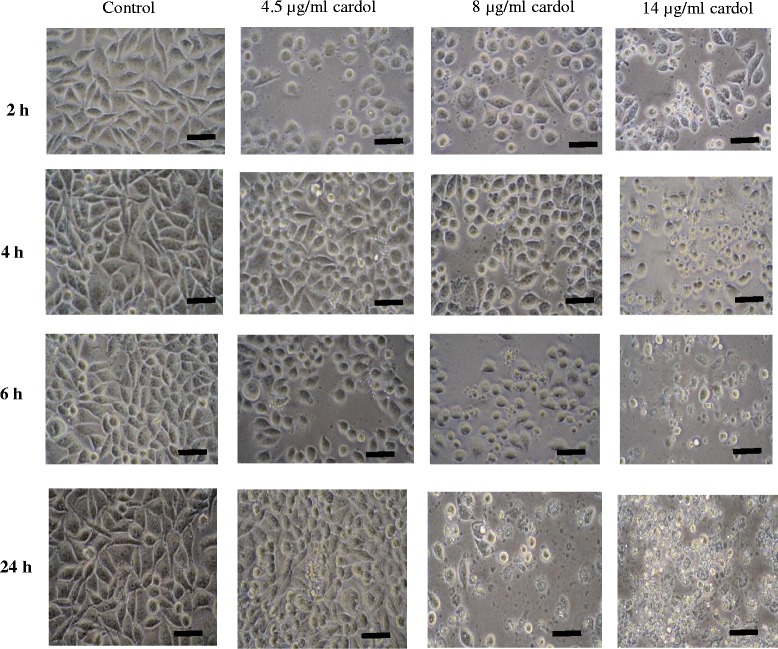
Morphology of SW620 cells at 2, 4, 6 and 24 h after treatment with cardol at 0 μg/mL (Control), 4.5 μg/mL (IC_50_), 8 μg/mL and 14 μg/mL. Images are at 400 x magnification and are representative of those seen from at least three such fields of view per sample and three independent repeats. Scale bar = 50 μm

The morphology of SW620 cancer cells after treatment with cardol at 4.5 μg/mL (IC_50_), 8 μg/mL and 14 μg/mL, was observed under a light microscope, with representative examples shown in Fig. 1. The shape of the SW620 cells had changed after 2 h treatment (the earliest time point examined) with cardol, with apoptotic and bulb appearing cells being evident.

**Fig. 2:**
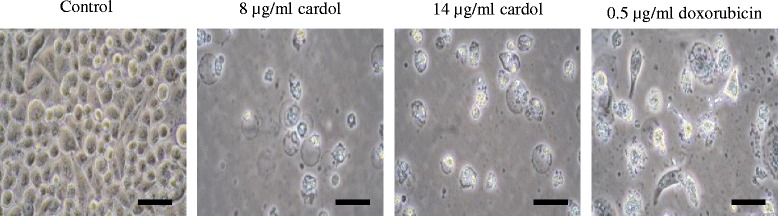
Morphology of SW620 cells at 72 h after treatment with cardol at 0 μg/mL (control), 8 and 14 μg/mL, or doxorubicin at 0.5 μg/mL. Images are at 400 x magnification and are representative of those seen from at least three such fields of view per sample and three independent repeats. Scale bar = 50 μm

Compared to the control, after 72 h of treatment very few SW620 cells treated with cardol at 8 or 14 μg/mL or doxorubicin (a common therapeutic drug for cancer) at 0.5 μg/mL had survived (Fig. 2). This indicated the antiproliferative activity of cardol and that it might be a potential chemotherapy agent like doxorubicin.

### Effect of cardol on the caspase gene expression level

**Fig. 3:**
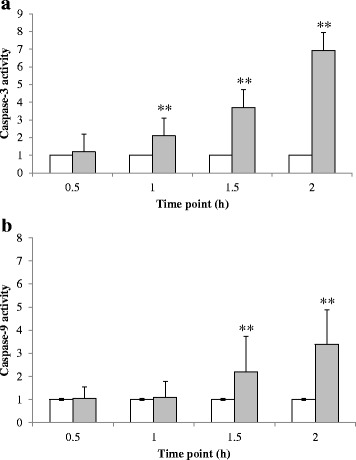
Effect of cardol treatment at 14 μg/mL on the activity of (**a**) caspase-3 and (**b**) caspase-9 in SW620 cells. White bars represent the control and grey bars are the cardol-treated cells. Data are expressed as the mean ± SE, derived from three independent repeats. Compared to the control, ** represent a significant difference at the *p* < 0.01 level

The activity of caspase-3 and caspase-9 in SW620 cells treated with cardol at 14 μg/mL was measured every 30 min for 2 h. The activity of caspase-3 and -9 were both increased in a time-dependent manner, being significantly higher than in the control after 1 h and still increasing over the second hour of treatment (Fig. 3).

**Fig. 4:**
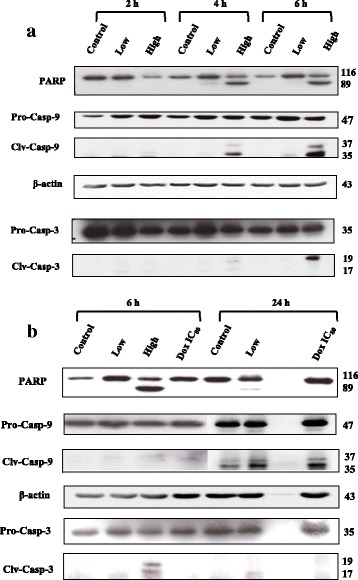
Representative western blot showing the effect on SW620 cells of cardol at 0 μg/mL (control), 8 μg/mL (Low) and 14 μg/mL (High) on caspase protein expression levels at (**a**) 2, 4 and 6 h after treatment and (**b**) compared to that with doxorubicin (Dox) at the IC_80_ dose (1.01 μg/mL) at 6 and 24 h after treatment. Pro- and cleaved (clv) forms of protein bands are shown. The figures are representative of five replications

Accordingly, the cleavage of PARP and caspase-3, as a hallmark of apoptosis, was evaluated by western blot analysis, while caspase-9 cleavage was used to indicate the involvement of the mitochondrial apoptotic pathway. The expression level of these proteins at 2, 4, 6 and 24 h after treatment with cardol at 0 (control), 8 and 14 μg/mL is shown in Fig. 4, where an effect of cardol at 14 μg/mL was observed from 4 and 6 h after treatment (24 h is not shown as most cells were dead).

Comparison of the effect of cardol treatment on SW620 cells with that of doxorubicin at the IC_80_ dose (1.01 μg/mL) after 6 h treatment revealed that cardol triggered cleavage of PARP, caspase-3 and caspase-9, but doxorubicin did not (Fig. 4b).

### Effect of cardol on the mitochondrial membrane potential

**Fig. 5:**
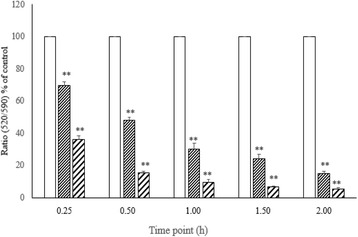
Effect of cardol on the mitochondrial membrane potential of SW620 cells. The mitochondrial membrane potential of SW620 cells treated with cardol at 0 μg/mL (Control, white bar), 8 μg/mL (small striped bar) and 14 μg/mL (large striped bar) was evaluated by JC-10 staining every 15 min for 2 h. Data are expressed as the mean ± SE, derived from four independent repeats. All cardol treatments were significantly different (*p* < 0.01) from the control and each other at each time point

Mitochondria are involved in the central pathway of multiple cell death mechanisms. Accordingly, the mitochondrial membrane potential was monitored using the fluorescent JC-10 probe that passes through the mitochondrial membrane and its accumulation in mitochondrial matrix is maintained by the mitochondrial membrane potential. SW620 cells treated with cardol at 8 and 14 μg/mL showed a dose- and time-dependent reduction in the JC-10 probe (membrane potential), being detected from 15 min of treatment onwards (Fig. 5). The cardol-induced mitochondrial depolarization could subsequently trigger caspase-9 activation and so the loss of cell viabilty.

### Effect of cardol on the intracellular ROS levels

**Fig. 6:**
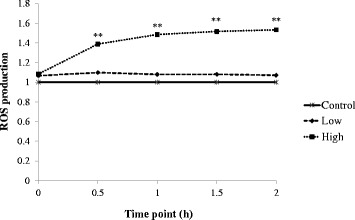
Effect of cardol at 0 μg/mL (Control), 8 μg/mL (Low) and 14 μg/mL (High) on intracellular ROS production, as determined by the DHE assay. Data are expressed as the mean ± SE, derived from three independent repeats. Compared to the control, ** represent a significant difference at the *p* < 0.01

Effect of cardol (0, 8 and 14 μg/mL) on the intracellular ROS levels in SW620 cells was measured every 30 min over a 2-h post-treatment period using the superoxide sensitive fluorescent probe DHE. Overall, cardol treatment of SW620 cells increased the DHE level after 30 min (Fig. 6), suggesting the increased production of ROS.

### The effect of cardol on ROS induction and cell death

**Fig. 7:**
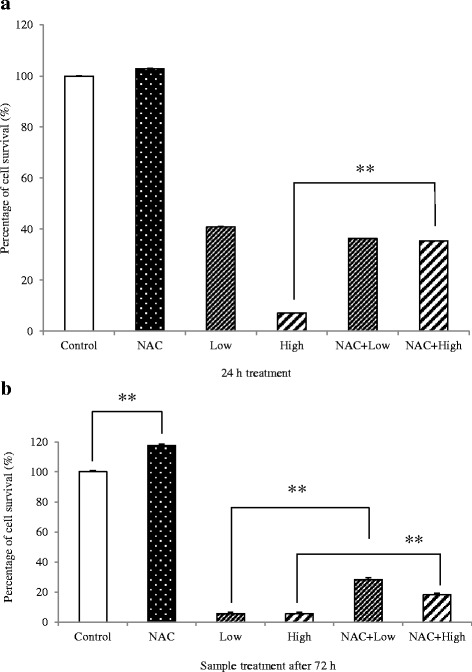
Effect of cardol at 0 μg/mL (Control), 8 μg/mL (Low) and 14 μg/mL (High), with or without 10 mM NAC, on the relative cell survival (%) after (**a**) 24 h and (**b**) 72 h exposure. A significant difference between the two indicated means (bar) is shown by ** for *p* < 0.01 Data are expressed as the mean ± SE, derived from three independent repeats

To investigate the potential role of the ROS elevation in cardol-induced cell death, the effect of the addition of NAC, an antioxidant, on the cardol-induced cell death was investigated in SW620 cells. SW620 cells were divided into six treatment groups that were incubated in CM with (i) DMSO at 0.1% (v/v) (control), (ii) 10 mM NAC, (iii) cardol at 8 μg/mL, (iv) cardol at 14 μg/mL, (v) cardol at 8 μg/mL plus 10 mM NAC and (vi) cardol at 14 μg/mL plus 10 mM NAC. After 24 and 72 h treatment, the relative cell survival (%) was evaluated by the MTT assay, with the results summarized in Fig. 7. Compared to the control, cardol treatment of SW620 cells markedly reduced the relative cell survival in a dose- and time-dependent manner, while the addition of NAC had no effect after 24 h but increased the relative cell survival after 72 h. The co-addition of NAC with cardol resulted in a significantly higher relative cell viability at both cardol concentrations and time points. Thus, NAC partially reduced the antiproliferation of cardol in SW620 cancer cells, and so implies a potential role for cardol-induced ROS production in the observed cardol-induced cell mortality.

### Potential mechanism of the cardol-induced cell death in SW620 cells

**Fig. 8:**
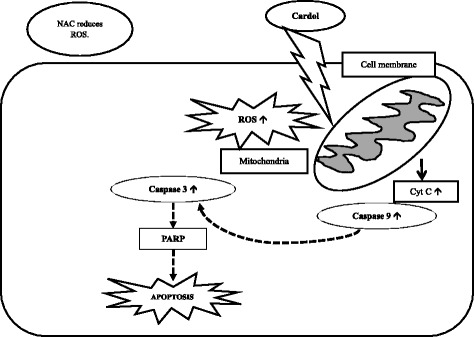
The proposed potential mechanism of the cardol-induced apoptotic pathway in SW620 cells

From the above data, a potential mechanism of the cardol-induced cell death in SW620 cells is proposed as schematically shown in Fig. 8.

## Discussion

Nowadays, there are increasing numbers of chemoresistant cancers to diverse chemotherapeutic drugs, while these drugs induce undesirable to severe side effects when used at their then required prolonged or high dose usage [14]. Thus, it is important to find an alternative agent, especially from natural products including propolis. Due to the variation in the composition of propolis, including its bioactive components, from different plant sources and biogeography, propolis has been classified into the seven groups of 1) poplar, 2) Brazilian green, 3) birch, 4) red, 5) Mediterranean, 6) clusia and 7) Pacific propolis [15–17]. The major bioactive compounds were found to be different among these seven different propolis groups. In addition to the type of the core compound structures, several other factors could affect the structure activity relationship of a compound (such as polymeric polyphenols or simple monomeric phenolics), including the number and position of the hydroxyl and methoxyl groups in the benzene ring and substituents that alter the possibility of electron delocalization in the double bonds [18]. Considering only Pacific propolis, Su et al. [19] reported the presence of propolin in Taiwanese propolis and ten phenylpropanoic acid esters were found in Korean propolis [20]. In addition, 11 compounds were reported from Indonesian propolis but, interestingly, not cardol [21]. In this research, we reported how cardol, also a major compound in the Indonesian propolis from at least *T. incisa*, could induce apoptosis in SW620 cells.

The morphological apoptotic characteristics, including cellular shrinkage, membrane blebbing and eventually fragmentation into membrane bound apoptotic bodies were observed after cardol treatment (Figs. 1 and 2) in a time-dependent manner. During apoptosis, the cell membrane loses its asymmetry exposing phosphatidylserine on the cell surface, which then functions as a destruction signal to macrophages to mediate the effective clearance of apoptotic cells [22].

After cardol-treatment the SW620 cells showed confounding factors, such as cell death, blebbing and loss of adhesion (Fig. 1), while cell rounding also occurred with cardol treatment at 8 μg/mL. Moskwa et al. [23] reported a similar observation of cell shrinking and rounding of the U87MG human glioblastoma multiform cell line after exposure to 1% and 2.5% (w/v) Polish honey. The morphological alteration suggested that the inhibition of (cancer) cell adhesion by honey might not be mediated through cytoskeletal rearrangement and, therefore, might be mediated by a receptor [24].

The morphological change in the cardol-treated SW620 cells seen here corresponded temporally with the cleavage of caspase-3 and PARP (Fig. 4). Furthermore, the cleavage of caspase-9 indicated a likely involvement of the mitochondrial apoptotic pathway in the cell death-inducing effect of cardol (Fig. 4). Activation of caspase-3 and caspase-9 was preceded by the disturbance of the mitochondrial membrane potential, which occurred in a time-dependent manner (Fig. 5), and by the generation of ROS in the cardol-treated SW620 cancer cells (Fig. 6). All the obtained data support the induction of SW620 cell death by apoptosis, which agrees with that reported previously [3, 25]. Although esculetin (a coumarin compound) blocked the cell cycle at the S subphase, it induced apoptosis in the SMMC-7721 human hepatocellular carcinoma cell line with a significant elevation in caspase-3 and caspase-9 activities [26].

The generation of ROS could be involved in initiating mitochondrial membrane permeabilization. Here, SW620 cells co-incubated with NAC, an antioxidant, were partially rescued from undergoing the cardol-induced apoptosis, and so ROS production may be involved in mediating cell death. In accord, ROS generation was detected in SW620 cells treated with cardol (Fig. 7). Similar to our work, the petroleum ether extract (PEE) of *Alpiniae oxyphyllae*, a member of the ginger (*Zingiberacea*) family, increased lactate dehydrogenase release, induced apoptosis, disrupted the mitochondrial membrane potential, and elevated intracellular ROS levels in HepG_2_ hepatocellular carcinoma cells [27]. The co-addition of NAC prevented the apoptosis induced by the PEE-mediated ROS generation in HepG_2_ cells by enhancing the *Bax*/*Bcl-2* ratio, increasing cytochrome c in the cytosol, and activating caspase-3/9 [27].

Many mechanisms have been proposed as to how an active compound might function in cancer cells. For example, chrysin, a major compound from *Apis mellifera* in Thailand, could sensitize A549 and HeLa human cancer cell lines to the apoptosis-inducing factor TRAIL by inhibiting STAT3 and downregulating Mcl-1 [28]. Although either tamoxifen or caffeic acid phenethyl ester (CAPE) have been found to be antiproliferative to many cancer cell lines, both also produced a synergistic antiproliferative effect on the MCF-7 breast cancer cell line [29]. The combination of tamoxifen and CAPE provoked the activation of caspase-9 that subsequently switched on downstream executioner caspases, including caspase-3, and led to the intrinsic pathway of apoptosis. Like in our research, cardol treatment also enhanced the activation of caspase-9 to promote caspase-3 activity and upregulated the cleavage of PARP to induce apoptosis. In addition, cardol induced an elevated ROS level in the mitochondria.

## Conclusions

Regarding the importance of alternative sources for cancer treatment, cardol may be a potential alternative antiproliferative agent against colon cancer. The results obtained in this study proposed a potential mechanism for the cardol-induced cell growth inhibition and induction of apoptosis through the mitochondrial apoptotic pathway.

## Abbreviations

DHE: Dihydroethidium
MTT: 3-(4,5-dimethyl-thiazol-2-yl)2,5-diphenyl-tetrazolium bromide
NAC: N-acetyl cysteine
ROS: Reactive oxygen species
